# Status and influencing factors of work stress among nurse managers in western China: a cross-sectional survey study

**DOI:** 10.1186/s12912-024-01731-4

**Published:** 2024-01-24

**Authors:** Zhuoxin Yang, Huaping Huang, Guirong Li

**Affiliations:** 1 School of Nursing, Chengdu Medical College, Chengdu, China; 2 Nursing Department of Mianyang Central Hospital, School of Medicine, University of Electronic Science and Technology of China, No.12, Changjia Alley, Jingzhong Street, Fucheng District, 621000 Mianyang, China

**Keywords:** Nurse manager, Organizational support, Work stress, Cross-sectional study

## Abstract

**Aims:**

Due to the nature of their work, nurses are more prone to experiencing psychological consequences than other healthcare workers. However, evidence on the emotional burden of nurse managers in China is limited. Furthermore, perceived organizational support has been approved that can affect mental health outcomes. Therefore, this study aimed to investigate the status quo and influencing factors of nurse managers’ work stress, and their possible relationship with perceived organizational support, which could further provide more countermeasures to safeguard their health.

**Methods:**

A cross-sectional online survey of 21 secondary and tertiary hospitals was conducted in a city in Sichuan province, Southwest China, using a convenience sampling method between October and November 2022. Data were collected using the general information questionnaire, the Chinese version of the Stress Overload Scale (SOS) and the perceived Organizational Support Scale (POSS). SPSS 23.0 was applied to analyze the data.

**Results:**

In total, 380 participants completed the survey. The median scores (interquartile range [IQR]) for SOS and POSS were 60.50 (50.00, 70.75) and 51.00 (44.00, 55.00), respectively. The work stress of the nurse managers was negatively correlated with perceived organizational support (*P* < 0.01). Multiple linear regression analysis showed that age older than 40 years, from secondary hospitals, working in emergency or pediatric wards, and professional qualification of supervisor nurse or deputy supervisor nurse, and the scores of POSS less than 51 significantly influenced the work stress of the nurse managers.

**Conclusions:**

Our study shows that nurse managers are more prone to work stress, and organizational support can effectively reduce this stress. Governments and hospital administrators should pay more attention to providing comprehensive strategies based on various risk factors to protect and promote psychological health.

## Introduction

The 2023 World Health Organization (WHO) statistical report revealed that nurses play a critical role in achieving universal health coverage and sustainable development goals [1]. With the development of global health and safety, nurses are under increased occupational stress due to rapid changes in the healthcare system and the particular nature of nursing work [2–4]. Especially during the COVID-19 pandemic, the continuous crisis response to public health emergencies directly affected nurses’ physical and psychological health [5, 6]. Occupational stress was defined as the state of physical and mental stress that occurs as a result of the incongruity between situational demands and an individual's ability to meet all of these demands [7]. Nurse managers play a vital role in directing the functioning of a practice area, influencing work culture, processing interprofessional collaboration and creating positive patient care outcomes [8]. Therefore, nurse managers are highly susceptible to emotional and physical exhaustion because they are constantly exposed to major stressors, such as time pressure, demanding obligations, workloads, etc. [9]. which resulted in an increasing number of nurse managers leaving or intending to leave their current job [10]. Reducing the work stress of nurse managers to improve job satisfaction is key to retain these individuals in their roles [11].

Perceived organizational support refers to the perceptions and beliefs of employees about how the organization views their contributions and cares about their well-being [12]. Previous studies have demonstrated that organizational support affected nurses’ professional identity and had a positive influence on improving job satisfaction and reducing intention of turnover [13, 14]. A recent study by Chatzittofis et al. [15] showed that perceived organizational support was significantly associated with the self-assessed mental status of healthcare workers. Furthermore, another study [16] explored the relationship between work stress and perceived organizational support in young Korean nurses and the results revealed that perceived organizational support can relieve early-to-mid-career nurses’ work stress, thus facilitating patient-centered care. However, little is known about its effects on reducing occupational stress in nurse managers. Consequently, this study aimed to (1) evaluate the status quo and its related factors of work stress, and (2) explore the correlation between work stress and perceived organizational support.

## Materials and methods

### Study design and participants

This cross-sectional study was conducted between October 2022 and November 2022. A convenience sampling method was used to select participants in 21 hospitals, involving 13 tertiary hospitals and 8 secondary hospitals, from a city in Sichuan Province, southwest China. All participants met the following inclusion criteria: (1) had served as nurse managers for more than one year, (2) were engaged in clinical front-line management work, and (3) had signed informed consent and volunteered to participate in this study. Participants who took less than 3 min to complete the questionnaires were excluded. A priori power analysis was performed using the G^*^power 3.1.9.7 software [17], where the required sample size was calculated by using F tests and linear multiple regression: fixed model, R^2^ deviation from zero as the statistical test, and ‘A priori: compute required sample size—given α, power, and effect size’ as the type of power analysis. Cohen’s f^2^ = 0.15 with medium effect size, α = 0.05, power (1 − β) = 0.80, and 25 predictors were taken in the model. The result of the analysis indicated that the minimum required sample size was 172. Meanwhile, considering the non-response rate might be 10%. Finally, the total number of participants was determined to be 189.

In this study, the questionnaires were imported into the online platform WenJuanXing (https://www.wjx.cn/) and sent to hospitals through the nursing society and the nursing quality control center. A uniform guideline was used to inform participants about the purpose and background of this study and its confidentiality measures before the survey. Nurse managers who were interested in this survey participated and completed the questionnaires using a computer or smartphone by opening a website link or scanning a quick response code. All the questions in the survey were set as mandatory, so participants who did not complete all the questions could not submit them. The same IP address was only filled once.

### Measurements

#### General information questionnaire

The research team designed the questionnaire based on a literature review, including gender, age, marital status, number of births, educational level, hospital grade, working wards, professional qualifications, position, professional experience (years) and professional experience (years).

#### The Chinese version of stress overload scale (SOS)

This scale was developed by Amirkhan [18] and translated and introduced by Su et al. [19] in 2014. The scale consists of two dimensions, namely event load and individual vulnerability, involving 22 items, using the Likert 5-level scoring method. Scores are calculated from 1 to 5 points for “never before” to “always there,” with a total score ranging from 22 to 110 points. The higher the score, the greater the stress load. Total scores of the two dimensions were placed in four quadrants (low pressure, susceptibility, shock, and high pressure) and three risk levels (minimum risk, low risk, and high risk). Cronbach’s α coefficient of reliability was 0.936, and this study achieved a reliability coefficient of 0.953.

#### Perceived organizational support scale (POSS)

The POSS was revised and applied by Zuo [20] in 2009 to evaluate subjective perceived organizational support among nurses. The scale includes 2 dimensions with a total of 13 items. The scale was scored on a 5-point scale ranging from ‘1’ very non-conforming to ‘5’ (very conforming). Higher scores indicated stronger organizational support. The Cronbach’s α coefficient of reliability of the original scale was 0.90, while this study had a reliability coefficient of 0.92.

### Data analysis

SPSS software version 26.0 (IBM Corp) was applied for data statistical analysis. Binary variables were expressed as numbers with percentages. Continuous variables were presented as medians with interquartile ranges (IQR) because the Kolmogorov-Smirnov test showed that the quantitative data exhibited a skewed distribution. The nonparametric Mann–Whitney U test and Kruskal–Wallis H test were used for intergroup comparisons. Spearman’s correlation analysis was used to analyze the relationship between work stress and perceived organizational support. A multivariate linear regression analysis was conducted to explore the influence of various factors on work stress. In the present study, regardless of the univariate correlation with work stress, all demographic parameters and the scores of POSS scores were initially included in the model. The coefficient of determinations such as R^2^, and the amount of variance, etc. were used to detect the effect size. *P* < 0.05 was considered statistically significant.

### Ethical considerations

This study was reviewed and approved by the Medical Ethics Committee of Mianyang Central Hospital, School of Medicine, University of Electronic Science and Technology of China and complied with the Strengthening the Reporting of Observational Studies in Epidemiology (STROBE) guidelines [21].

## Results

### Sociodemographic characteristics of nurse managers

Table 1 shows the baseline sociodemographic characteristics of the nurse managers. In total, 380 nurse managers completed the survey. Of these, 371 (97.63%) were female, 212 (55.79%) were 31–40 years old, 150 (39.47%) were over 40 years old, 236 (62.11%) worked in tertiary hospitals, 239 (62.89%) were nurse managers with professional qualifications of supervisor nurses and 343 (90.26%) were head nurses.

**Table 1:** Sociodemographic characteristics of nurse managers (*N* = 380)

Demographics	N (%)	SOS total scores^*^	*P* value
**Gender**			0.63^#^
Male	9 (2.37)	62.00 (50.33–75.00)	
Female	371 (97.63)	60.00 (50.00–70.00)	
**Age (years)**			0.47^†^
20 ~ 30	18 (4.74)	62.50(46.83–77.17)	
31 ~ 40	212 (55.79)	62.00(50.00–70.58)	
> 40	150 (39.47)	59.00(47.00–70.00)	
**Marital status**			0.73^†^
Yes	347 (91.32)	60.00 (49.00–71.00)	
No	16 (4.21)	62.50 (46.42–71.17)	
Divorce	17 (4.47)	64.00 (56.00–68.00)	
**Number of births**			0.40^†^
None	23 (6.05)	63.00 (46.17–71.67)	
One	249 (65.53)	60.00 (47.00–69.00)	
Two	108 (28.42)	61.00 (53.00–71.58)	
**Educational level**			0.86^†^
Below undergraduate	71 (18.68)	58.00 (48.33–72.67)	
Undergraduate	301 (79.21)	61.00 (49.67–70.00)	
Postgraduate	8 (2.11)	62.00(59.67–67.50)	
**Hospital grade**			0.78^†^
Tertiary	236 (62.11)	61.00 (49.42–70.00)	
Secondary	144 (37.89)	59.00 (50.00–71.00)	
**Wards**			0.13^†^
Surgical	150 (39.47)	58.00 (47.92–70.00)	
ICU	19 (5)	58.00 (45.00–75.33)	
Internal	82 (21.58)	63.00 (53.00–71.08)	
Nursing department	39 (10.26)	59.00 (51.33–62.83)	
Emergency	37 (9.74)	66.00 (56.67–74.33)	
Operation room	22 (5.79)	57.00 (49.75–63.00)	
Pediatric	31 (8.16)	63.00 (47.67–73.33)	
**Professional qualifications**			0.25^†^
Junior nurse	53 (13.95)	57.00 (45.67–64.67)	
Supervisor nurse	239 (62.89)	62.00 (50.00–71.00)	
Deputy chief nurse	77 (20.26)	60.00 (52.67–72.33)	
Chief nurse	11 (2.89)	58.00 (50.17–65.17)	
**Position**			0.25^#^
Head nurse	343 (90.26)	61.00 (49.00–71.00)	
Director	37 (9.74)	59.00 (53.00–63.00)	
**Professional experience (years)**			0.75^†^
< 5	4 (1.05)	68.00 (51.33–76.50)	
5 ~ 10	37 (9.74)	62.00 (50.67–67.33)	
> 10	339 (89.21)	60.00 (49.17–70.83)	
**Management experience (years)**			0.48^†^
< 5	150 (39.47)	61.00 (50.00–69.08)	
5 ~ 10	128 (33.68)	60.50 (50.00–72.58)	
> 10	102 (26.84)	60.00 (46.83–69.08)	
**Total scores for POSS**			< 0.001^#^
> 51	174 (45.8)	54.00 (45.00–64.00)	
≤ 51	206 (54.2)	64.00 (57.00–74.00)	

Note: IQR = interquartile range; ICU = intensive care unit; POSS = perceived organizational support scale; SOS = stress overload scale.

^*^Presented as median (IQR, interquartile range).

^#^ Mann-Whitney U test.

^†^ Kruskal-Wallis H Test.

### Stress load and perceived organizational support of nurse managers

The results showed that the median (IQR) score of stress load was 60.50 (50.00, 70.75) points. Of which, the event load score was 34.00 (29.00, 38.00) points and the personal vulnerability score was 26.00 (22.00, 33.00) points, indicating low-risk shock work stress. The median (IQR) score of POSS was 51.00 (44.00, 55.00) points.

### Correlations between stress burden and perceived organizational support among nurse managers

The findings of Spearman’s correlation analysis showed that the total scores for SOS and its two dimensions were negatively associated with the total scores for POSS (*P* < 0.01), indicating that the higher the perceived organizational support scores, the lower the stress load. See Table 2.

**Table 2:** Spearman’ s correlation analyses between perceived organizational support and work stress (*N* = 380)

Variables	Total scores for POSS	Emotional Support	Instrumental support
Total scores for SOS	-0.381**	-0.377**	-0.348**
Event load	-0.322**	-0.324**	-0.279**
Personal vulnerability	-0.385**	-0.376**	-0.363**

Notes: POSS, perceived organizational support scale; SOS, Stress overload scale; ^**^P < 0.01.

### Risk factors associated with work stress in nurse managers

Table 3 presents the results of the multiple linear regression analysis of the influencing factors of work stress. The findings indicated that age older than 40 years (β = -9.01, 95% CI = -17.14 to -0.88), from secondary hospitals (β = 3.41, 95% CI = 0.58 to 6.23), work in emergency (β = 6.67, 95% CI = 2.17 to 11.18) or pediatric ward (β = 5.13, 95% CI = 0.26 to 10.01), with the professional qualification of the supervisor nurse (β = 7.89, 95% CI = 3.59 to 12.19) or the deputy supervisor nurse (β = 11.68, 95% CI = 6.22 to 17.14) and POSS total scores less than 51 (β = 7.92, 95% CI = 5.21 to 10.62) affected the work stress of nurse managers. These variables only explained 14.00% of the variance between the dependent and independent variables (R^2^ = 0.14, F = 2.34, *P* < 0.001).

**Table 3:** Multiple linear regression results of the influencing factors of work stress (*N* = 380)

Variables		Unadjusted coefficients	adjusted coefficients
		B (95% CI)	Beta (β) (95% CI)
**Gender** (vs. male)	Female	-2.23 (-10.99 to 6.54)	-1.43 (-10.29 to 7.44)
**Age** (years, vs. 20 ~ 30)	31 ~ 40	-2.14 (-9.79 to 5.51)	-6.12 (-13.94 to 1.71)
	> 40	-3.81 (-11.53 to 3.90)	-9.01 (-17.14 to -0.88)
**Marital status** (vs. yes)	No	0.32 (-6.33 to 6.97)	-2.71 (-13.56 to 8.13)
	Divorce	2.37 (-4.10 to 8.83)	2.09 (-4.39 to 8.57)
**Number of births** (vs. None)	One	-1.23 (-6.89 to 4.43)	-0.09 (-9.62 to 9.43)
	Two	0.70 (-5.27 to 6.66)	0.92 (-8.52 to 10.35)
**Educational level** (vs. below undergraduate)	Undergraduate	0.35 (-3.09 to 3.78)	-0.02 (-3.86 to 3.83)
	Postgraduate	1.89 (-7.81 to 11.60)	2.39 (-7.84 to 12.63)
**Hospital grade** (vs. tertiary)	Secondary	1.06 (-1.67 to 3.78)	3.41 (0.58 to 6.23)
**Wards** (vs. surgical)	ICU	-5.72 (-12.74 to 1.30)	-4.42 (-11.34 to 2.50)
	Internal	2.32 (-1.09 to 5.73)	2.50 (-0.90 to 5.90)
	Nursing department	-2.02 (-6.49 to 2.45)	-3.47 (-8.20 to 1.25)
	Emergency	6.05 (1.48 to 10.62)	6.67 (2.17 to 11.18)
	Operation room	-3.53 (-9.32 to 2.26)	-3.26 (-9.04 to 2.52)
	Pediatric	4.68 (-0.25 to 9.62)	5.13 (0.26 to 10.01)
**Professional qualifications** (vs. junior nurse)	Supervisor nurse	4.82 (0.78 to 8.86)	7.89 (3.59 to 12.19)
	Deputy chief nurse	5.87 (1.21 to 10.53)	11.68 (6.22 to 17.14)
	Chief nurse	0.85 (-8.12 to 9.83)	6.29 (-2.79 to 15.37)
**Position** (vs. head nurse)	Director	-2.48 (-6.97 to 2.01)	1.72 (-7.80 to 11.23)
**Professional experience** (years, vs. < 5)	5 ~ 10	-4.26 (-17.95 to 9.44)	-2.28 (-16.35 to 11.79)
	> 10	-4.53 (-17.61 to 8.56)	-1.56 (-15.59 to 12.47)
**Management experience** (years, vs. < 5)	5 ~ 10	0.69 (-2.43 to 3.81)	0.03 (-3.34 to 3.40)
	> 10	-1.76 (-5.09 to 1.57)	-2.01 (-6.35 to 2.33)
POSS scores (vs. >51)	≤ 51	8.07 (5.53 to 10.61)	7.92 (5.21 to 10.62)

Notes: R^2^ = 0.14, F = 2.34, *P* < 0.001; CI = Confidence Interval; ICU = intensive care unit; POSS = perceived organizational support scale.

## Discussion

### Analysis of the current state of work stress and perceived organizational support among nurse managers

The results showed that the total stress load score of the nurse managers was 60.5 (50.00, 70.75), indicating that this group is more likely to be at risk of developing stress-related illnesses, which is similar to the survey results of Gu et al. [22]. The event load score in this study was higher than that reported in previous studies, the possible reason is that the participants in this study were nurse managers, who were clinical nursing practitioners and nursing decision-makers and were required to have more solid professional knowledge and the ability to handle matters. At the same time, the survey period was during the COVID-19 pandemic, when the demand for nursing human resources in the society rose sharply, the number of people working in hospitals decreased, and the workload increased sharply. Nurse managers should not only coordinate their work but also balance human resources and scheduling and be under significantly higher pressure than ordinary clinical nurses. Research has found that scientific stress management methods and psychological counseling can alleviate nurses’ job burnout and negative emotions, reduce the incidence of adverse events, and thus enhance their professional identity [23]. Therefore, hospitals should strengthen the ability to manage stress and psychological counseling training of nurse managers and allocate personnel reasonably through learning flexible shift adjustment and increasing the number of “mobile nurse libraries” [24]. Attention must be paid to the physical and mental state of the nurse managers, and early detection, early intervention, and early counseling must be performed for those with moderate to high-stress load levels, ultimately reducing their stress load.

The results showed that the total score of organizational support for nurse managers was 51.00 (10.00, 13.00), which was above the average level. Among them, the score for the emotional support dimension was relatively low, indicating that the hospital provided insufficient emotional support, which is consistent with previous research results [25]. The organizational support score in this study was higher than that of another study [26], which may be related to the research subjects and the period of the survey. The research participants in this study were nurse managers, and the organization provided them with more material and spiritual support than ordinary nurses. During the survey period, the country and society attached great importance to nursing work [27], which made them feel more valued, recognized, and cared for by the organization, indirectly increasing the professional identity and organizational support of nurse managers, resulting in higher scores of organizational support. According to the social exchange theory, both parties in the exchange relationship follow the “principle of reciprocity“ [28]. When employees feel the support and trust of the organization in their work, they feel that their work is full of value and achievements, which increases their sense of professional mission, and they work more actively. Therefore, hospitals should provide material and spiritual support to nurse managers, meet their self-actualization needs, and encourage groups to exert subjective initiative, thereby increasing their sense of organizational support [29].

### The relationship between perceived organizational support and work stress among nurse managers

The results showed a negative correlation between perceived organizational support and work stress (*P* < 0.01), which is consistent with another research results [16]. When nurse managers do not feel the support and affirmation of the organization, they can question and feel powerless about their work and doubt its value [30]. Ultimately, work pressure cannot be alleviated. Research has indicated that a high level of organizational support can help improve employees’ subjective initiative and enthusiasm for work, and this positive emotion can help them overcome difficult environments, alleviate the pressure generated in clinical nursing work, and reduce perceived pressure [23]. According to the theory of self-determination [31], employees feel empowered by the organization, which not only meets their power and achievement needs, but also motivates their autonomy, ultimately enhancing their perception of internal identity. It is recommended that hospitals actively understand the deep-seated needs of nurse managers, provide sufficient resources and support in the work process, and promote the role of nurse managers in the development of hospitals and the maintenance of patient health; pay attention to the mental health of nurse managers, strengthen effective communication, and promote their stress release. Hospitals should also delegate power and be adept at delegating power in order to provide a platform for nurse managers to fully exercise their autonomy and enhance their sense of organizational support [30].

### Risk factors associated with work stress of nurse managers

This study showed that the older the age, the lower the stress load and the greater the sense of organizational support among nurse managers, which is consistent with a previous research result [32]. As managers age, their work experience, quality, and efficiency increase, and they can handle work tasks effortlessly. Consequently, their organizational benefits and sense of professional belonging increase, promoting their sense of organizational support. Therefore, hospitals should increase their leadership training for young nurse managers, improve their job competency and self-efficacy, establish and encourage them to participate in psychological activity groups, and exercise their excellent professional ability and psychological quality; communication should be promoted among nurse managers through hospital cultural construction, such as establishing a balanced group to strengthen communication between doctors, nurses and patients, and share work experience. The importance of education of professional values for young nurse managers should be emphasized, while providing timely recognition and rewards for their work and enhancing their sense of organizational support [29].

Differences in pressure among nurse managers with different professional qualifications. With an increase in professional titles, the pressure load shows a trend of initially increasing and then decreasing, which may be related to the previous professional title promotion system for health technicians in China [33]. As leaders of the nursing team, nurse managers perform multiple functions, such as administration and business management. Supervisor nurses and deputy chief nurses have been working on the front line for a long time and their problem-solving skills are still insufficient. In addition, competition for the promotion of professional qualificationsis fierce, putting heavy pressure on the group [34]. The chief nurse is the highest professional title and there is no pressure for promotion. Fatigue often occurs after work, and lack of attention to improving one’s abilities leads to decreased stress levels. Therefore, hospitals should conduct hierarchical management based on different professional titles, increase training for nurse managers with lower professional titles, and promote the improvement of their comprehensive quality; the management of nurse managers with senior professional titles should be strengthened after their appointment by refining assessments [35], avoiding the “one size fits all” approach to professional titles, and mobilizing their work enthusiasm; a team support system should be built, such as organizing employee assistance plans to promote the dissemination of higher vocational titles by managers with lower vocational titles and play a role in spreading, helping and guiding; a sound reward and punishment mechanism should be established to promote work enthusiasm and organizational support of nurse managers in the entire hospital, effectively improve the overall quality of nursing, and ultimately achieve a dynamic balance between pressure of nurse managers and organizational support.

This study suggested that the higher the hospital grade, the greater the stress load on nurse managers and the stronger their sense of organizational support, which is consistent with previous research results [36]. Perhaps because the higher the hospital grade, the richer the medical facilities and nursing resources and the more visits, nurse managers must have a more abundant clinical knowledge reserve and practical skills. Therefore, the higher the hospital level, the higher the stress load on nurse managers, and the higher the importance that hospital management attaches to nursing, the stronger the sense of organizational support it provides. Secondary hospitals have a low sense of organizational support, which may be due to the hospital placing more emphasis on the overall development of the hospital and insufficient emphasis on the nursing team, resulting in a low sense of organizational support [36]. Nurses have the largest workload and are closest to patients in hospitals. The quality of their work directly affects the quality and social image of hospitals. Hospital managers should emphasize the importance of the nursing team and provide appropriate organizational support for nurse managers to reduce work pressure and improve professional identity and work enthusiasm [27].

The working ward with the highest stress load in this study was the emergency department. The nature and working environment of different departments can affect the pressure and organizational support of nursing staff [37]. From the perspective of sudden public health emergencies, emergency medical staff on the front line of hospitals face higher occupational exposure risks, work overload, and serious psychological impact. Therefore, hospitals should fully assess the risks, pressures, and management difficulties faced by different departments and provide targeted organizational support, such as rational allocation of human resources, increased psychological support and counseling, organizational culture and emotional support, and increased team-building opportunities for departments with low organizational support. Promotion of participation of nurse managers in decision making through authorized leadership can help build a harmonious hierarchical relationship, allowing them to feel trust and support from the organization and ultimately strengthening their sense of organizational support [38].

## Conclusions

The results of this study showed that the stress load of nurse managers was moderate and there was a negative correlation between the stress load of nurse managers and the current state of organizational support. Therefore, hospitals must be of importance to pressure on nurse managers and provide targeted organizational support for different populations and types of stress to achieve a balanced development of the nursing management team in the entire hospital. This study carried out during the period of the Covid-19 pandemic and the results may provide a reference for actively responding to major public health emergencies in the future. The limitation of this study is that it only selected nurse managers from second-level hospitals and above in a certain city in Sichuan Province, which has the problem of insufficient representativeness of the results. In the future, multicenter and longitudinal studies should be conducted to explore more fully the factors affecting the stress load of nurse managers and develop targeted intervention measures.

## Data Availability

The datasets collected and analyzed in this study are available from the corresponding author upon reasonable request.
